# Neural mechanisms for turn-taking in duetting plain-tailed wrens

**DOI:** 10.3389/fncir.2022.970434

**Published:** 2022-09-23

**Authors:** Melissa J. Coleman, Nancy F. Day, Eric S. Fortune

**Affiliations:** ^1^W.M. Keck Science Department, Claremont McKenna, Scripps and Pitzer Colleges, Claremont, CA, United States; ^2^Department of Psychology, Whitman College, Walla Walla, WA, United States; ^3^Department Biological Sciences, New Jersey Institute of Technology, Newark, NJ, United States

**Keywords:** antiphonal, birdsong, duets, central pattern generator, auditory feedback, neuroethology

## Abstract

Recent studies conducted in the natural habitats of songbirds have provided new insights into the neural mechanisms of turn–taking. For example, female and male plain–tailed wrens (*Pheugopedius euophrys*) sing a duet that is so precisely timed it sounds as if a single bird is singing. In this review, we discuss our studies examining the sensory and motor cues that pairs of wrens use to coordinate the rapid alternation of syllable production. Our studies included behavioral measurements of freely–behaving wrens in their natural habitat and neurophysiological experiments conducted in awake and anesthetized individuals at field sites in Ecuador. These studies show that each partner has a pattern-generating circuit in their brain that is linked *via* acoustic feedback between individuals. A similar control strategy has been described in another species of duetting songbird, white–browed sparrow–weavers (*Plocepasser mahali*). Interestingly, the combination of neurophysiological results from urethane-anesthetized and awake wrens suggest a role for inhibition in coordinating the timing of turn–taking. Finally, we highlight some of the unique challenges of conducting these experiments at remote field sites.

## Introduction

**Figure 1:**
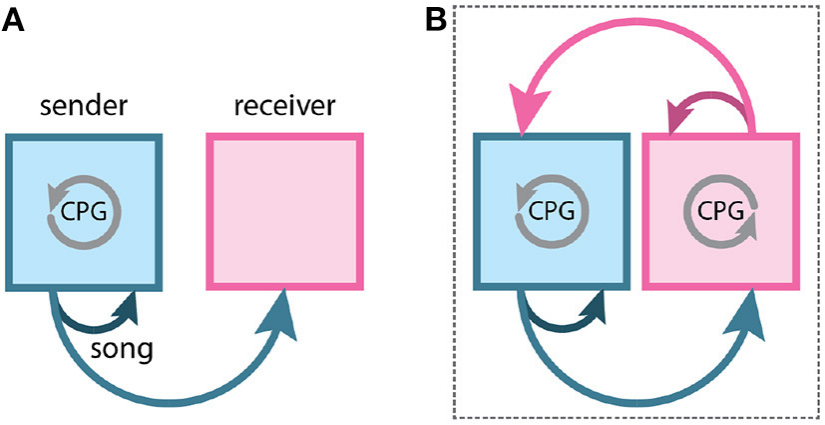
Social behaviors require communication between two animals. **(A)** Most neural studies of song control in songbirds focus on species in which only the male sings. In these birds, the pattern for the song is generated by a central pattern generator (CPG) in the male's brain (gray circular arrow). The song is then heard by the female (lighter-blue arrow), the receiver. The song is also heard by the male (dark blue arrow), which is required for song maintenance. **(B)** Turn-taking in wrens requires the exchange of information between a female and male. In duetting birds, singing is controlled by a CPG in the brain of each individual. The song of one individual is heard by the other and modulates the ongoing behavior of the partner. Thus, the two animals form a single circuit for the control of turn–taking (dotted box).

Social behaviors rely on sensory signals sent between a “sender” and a “receiver”. Signals produced by the sender often modulate the behavior of the receiver. For example, in most songbird species, males broadcast a song that may be heard by conspecifics (Figure 1A). These males produce their songs with at least two social goals – to attract females for reproduction and to repel competing males (Catchpole and Slater, 2018). When conspecifics respond to these songs, the sender/receiver relationship often reverses. If a male is the sender when producing its song to attract a female, it becomes the receiver when the female approaches it and produces a copulation solicitation display (Elie et al., 2019; Perkes et al., 2019). Indeed, in many social behaviors, individuals are constantly switching between roles as sender and receiver (Baker et al., 2019).

In turn–taking, a social behavior in which participants alternate behaviors, there is a rapid switching of sender and receiver roles in each participant (Pika et al., 2018; Elie et al., 2019; Banerjee and Vallentin, 2022). This alternation highlights a commonly overlooked feature of social behavior; when both participants act as both sender and receiver, an emergent feedback loop can form (Fortune et al., 2011; Coen and Murthy, 2016; Coleman and Fortune, 2018; Coleman et al., 2021) (Figure 1B). This feedback loop is mediated by the sensory signals that link activity in the brains of each participant. There has been a growing appreciation of the back-and-forth exchange of acoustic information between females and males across songbird species: both sexes are believed to have sung in the common ancestor of songbirds (Odom et al., 2014). Therefore, this form of emergent feedback loop for turn–taking is likely present in the common ancestor of songbirds.

How does neurophysiological activity in each participant generate tightly coordinated turn-taking? Tightly-coordinated duet singing in songbirds, such as plain-tailed wrens (*Pheugopedius euophrys*) and white–browed sparrow–weavers (*Plocepasser mahali*), are particularly well–suited for the study of the neural mechanisms of turn-taking (Brenowitz, 2021). Remarkably, we are aware of only three studies that examine the neural mechanisms for the control of duetting in songbirds (Fortune et al., 2011; Hoffmann et al., 2019; Coleman et al., 2021).

**Figure 2:**
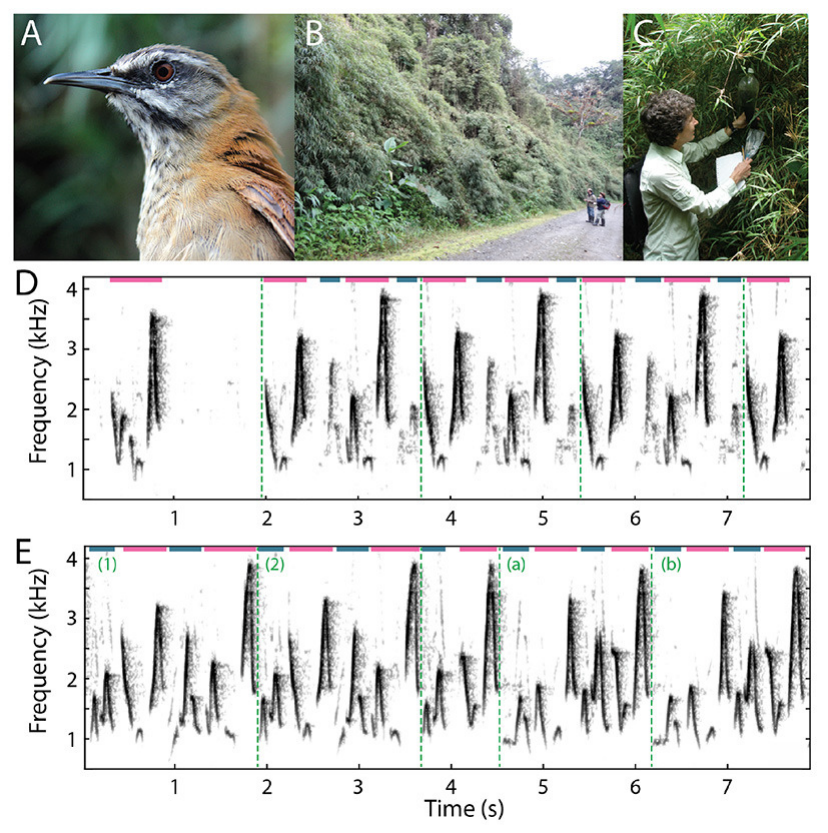
Field recordings of plain-tailed wren songs. **(A)** Photo of a plain-tailed wren. **(B)** Plain-tailed wrens live in dense bamboo on the slopes of the Andes. Photo taken near the Yanayacu Biological Field Station and Center for Creative Studies near Cosanga, Ecuador. **(C)** Setting up microphones in the bamboo to record wren duets. The microphones were covered with 2L soda bottles to protect them from rain. **(D)** Spectrogram of the beginning of a plain-tailed wren duet captured in the field. The female first sang a syllable as part of her solo song, then the male joined the duet. In this example, the male was further away from the microphone, so his syllables are of lower amplitude and can therefore be more readily distinguished from the female syllables. Female syllables are denoted with light magenta lines, and male with dark blue lines at the top of the spectrogram. Motifs (repeated sequences of syllables) are distinguished by vertical dashed green lines. **(E)** Segment of a song recorded in the field in which the structure of the duet changed. The first two motifs (1,2) are the same, then the birds produced a shortened motif around 4 sec of this recording. After the shortened motif, the pair sung a different motif (a,b). Syllables and motifs are labeled as in **(D)**. Spectrograms were rendered in Matlab (MathWorks) using the spectrogram function (95% overlap, either 512- or 1,024-point window, sample rates of 10 or 25 kHz).

In this review, we focus on our work with plain-tailed wrens (Figure 2A; Fortune et al., 2011; Coleman et al., 2021). These birds live in thick bamboo on the slopes of the Andes in Ecuador (Figure 2B), between 2200–2400 meters above sea level. Female and male pairs of birds sing a learned duet in which they rapidly (up to 6 Hz) alternate the production of their vocalizations so quickly and precisely (Figures 2D,E) it sounds as if a single bird is singing. Our behavioral and neurophysiological experiments were conducted at remote field stations in Ecuador, within the natural habitats of the animals. These field studies presented numerous challenges, both practical and scientific.

One interesting practical challenge that led to unexpected scientific insights was the choice of neurophysiological techniques. Initially we relied on recordings conducted in anesthetized wrens as these experiments seemed more feasible at our field sites (Fortune et al., 2011). Unexpectedly, these recordings in anesthetized birds were critical for the discovery of a likely role of inhibition for the coordination of duet singing in awake birds (Coleman et al., 2021).

### Background

Birdsong is a learned behavior that requires interactions between sensory and motor information in the brain (Mooney, 2014). In songbirds, juveniles match their own vocal output to a memory of a tutor song (Konishi, 1965; Ikeda et al., 2020). Once birds learn their song, ongoing auditory feedback is necessary to maintain their vocalizations, as song degrades when birds are deafened (Nordeen and Nordeen, 1992; Doupe and Kuhl, 1999; Leonardo and Konishi, 1999; Brainard and Doupe, 2001; Horita et al., 2008). This auditory feedback modulates motor circuits in the brain that control singing (Roberts et al., 2017). The neural basis of these sensorimotor interactions has been primarily studied in species in which only the male sings, such as zebra finches (*Taeniopygia guttata*), white-crowned sparrows (*Zonotrichia leucophrys*), bengalese finches (*Lonchura striata*), and canaries (*Serinus canaria*). The key difference between songbirds in which only males sing and duetting species in which both females and males sing together is the rapid exchange of acoustic information between birds. This information exchange is used, at a minimum, for the coordination of the duet performance.

There are dedicated circuits in songbird brains, known as the “song system”, that have been shown to control song learning, production, and maintenance (Brainard and Doupe, 2002; Mooney, 2014). Specifically, a nidopallial area known as HVC (proper name) is a site of sensorimotor integration for song (Mooney and Prather, 2005; Roberts et al., 2017). HVC is necessary for song production (Nottebohm et al., 1976) and contains neurons that form the pattern-generating circuit for song. Stimulation of HVC can ‘reset' the temporal patterning of song (Vu et al., 1994) and cooling HVC can slow song production (Long and Fee, 2008). HVC neurons are not only active when the bird is singing but also when the bird hears playbacks of its own song (Margoliash, 1983; Margoliash and Konishi, 1985; Prather et al., 2008). Interestingly, in species in which only males sing, the temporal pattern of HVC neuron activity is nearly identical when the birds are singing and to playback to their song (Prather et al., 2008; Mooney, 2014). The similarity in sensory- and motor-related HVC activity makes it challenging to differentiate these two signals.

Duetting birds, in contrast, receive two categories of feedback. From the perspective of an individual bird, the duet is composed of two types of syllables, those that it produced (autogenous) and those produced by the partner (heterogenous). Any syllable produced by the bird must involve the generation of motor patterns in the brain for singing, and hearing its own vocal output (Figure 1B). In contrast, when the other bird sings, there is no motor output. This is where duetting birds offer an advantage–when one of the birds is singing, its HVC has a sensorimotor interaction that is presumably similar to that which occurs in non-duetting species of songbirds. But when the partner bird is singing, it only receives sensory feedback. Therefore, we can use these different forms of sensory feedback to help us understand how behaviors are coordinated between two individuals.

## Turn-taking and other behaviors in plain-tailed wrens

To understand behavioral rules used by duetting wrens, we placed microphones in the thick bamboo stands in which they live (Figures 2B,C; Fortune et al., 2011). The birds sing extremely loud duets – we measured over 80 dB SPL standing within a few meters of the birds. The duet durations ranged from a few seconds to over 2 minutes of continuous singing (as reported in Mann et al., 2006). Birds repeated short-duration motifs, typically less than 2 seconds, composed of four to six unique syllables (Figures 2D,E). Females and males alternated syllable production within each motif, although individuals occasionally dropped a syllable (see Figure 1C in Fortune et al., 2011). The patterns of syllables in duets often sound quite consistent. Visual inspection of spectrograms, however, show that individuals make small changes in acoustic features, particularly in the time-varying frequency within syllables, over the duration of duets (Figure 2E). These variations appear to add additional complexity to duet performances. Future experiments may examine the potential relevance of these variations to the animals, and test whether each bird is responding to changing acoustic dynamics in its partner's syllables.

These recordings also showed that wrens produce “solo” songs in which either the female or male sings by itself (Figure 2D; see Figure 1 in Fortune et al., 2011). When males sing alone, their syllables are generally produced at much lower amplitudes than when they sing in duets. We also found that the solo songs of both individual female and individual male wrens are composed of similar syllables and sequences of syllables as seen in duets (see Figure 1 in Fortune et al., 2011).

Plain-tailed wrens are not prolific singers, singing perhaps dozens of duets in a day. We observed differences in singing from day-to-day: on some days they produced very few or no songs, whereas on other days all pairs of birds in the area sang throughout the day. We do not know the causes of these variations in singing (weather, food availability, etc.), but the song-producing circuits in the brain are likely modulated by environmental cues.

We also observed that the patterns of duet singing sometimes shift during longer-duration duets, resulting in an overt change in the cadence of the song (Figure 2E). How and why birds make these changes is unknown. On one hand, these changes in cadence may be a form of error correction following a poorly produced syllable. Alternatively, these changes may be cues used in territorial defense, to reinforce pair bonds, or as a mechanism for sexual selection. We also observed differences in duetting between birds in their natural habitat and when in captivity. Pairs of wrens in captivity typically sang shorter duration duets, on the order of seconds, and rarely produced longer duets. As a result, our neurophysiological experiments focused on shorter duration duets, and have not addressed mechanisms that underlie changes in cadence found in some longer duets.

### Exchange of sensory information in solo and duet singing

In plain-tailed wrens, acoustic cues alone are sufficient for the coordination of duetting, as birds that are visually isolated from each other produce normal duets. Remarkably, on a few occasions captured birds that were held temporarily in cloth bags duetted with their uncaptured partners. Additionally, wrens can initiate duets with more than ten meters between the individuals. In the field, we have heard duets in which one of the birds is adjacent to our location but the other bird is deep in the bamboo (Figure 2D). Delays due to the speed of sound – 10 meters is traversed in about 30 ms – are obvious to human listeners and affect the timing of turn–taking during duets.

Acoustic cues from the partner can modulate the timing of the duet. We observed a difference between solo and duet songs in the timing of syllables (Fortune et al., 2011). In both females and males, solo songs showed greater variability in the intersyllable intervals when compared to duets (Fortune et al., 2011). This change likely results from hearing partner syllables during duet singing. These data suggest that each bird has a pattern-generating circuit in its brain that produces its song, as is typical in oscine passeriform birds, and that sensory cues from the partner modulate the temporal dynamics of this pattern-generating circuit (Figure 1B).

These behavioral observations provided the framework for our neurophysiological recordings. In short, duet singing is produced by central pattern-generators (CPGs) in each bird, and acoustic cues from the partner modulate the timing of these CPGs. From previous studies, we knew that HVC acts as a CPG for song production (Long and Fee, 2008) and that HVC neurons receive auditory input (Margoliash, 1983; Mooney et al., 2001; Coleman et al., 2007). We therefore focused on HVC for neurophysiological recordings.

## Neurophysiological mechanisms for the coordination of duet performances

To understand the neural mechanisms for duet singing, we used two categories of neurophysiological recordings: recordings in urethane-anesthetized birds (Fortune et al., 2011), and recordings in awake, singing wrens (Coleman et al., 2021). Based on extensive previous work in other songbird species, we expected substantial differences in the patterns of neurophysiological activity in these two conditions. Previous studies in which HVC recordings were made in urethane-anesthetized birds showed that HVC neurons are highly selective for the bird's own learned song and typically do not respond to conspecific vocalizations (Margoliash, 1986; Mooney, 2000; Mooney et al., 2001). In these experiments, the bird is anesthetized, an electrode is placed in HVC, and pre-recorded songs and other sound stimuli are repeatedly played to the bird. We therefore expected that in plain-tailed wrens, neurons in HVC would respond to the bird's own vocalizations; that is, HVC neurons in males would respond to male syllables and neurons in females would respond to female syllables.

**Figure 3:**
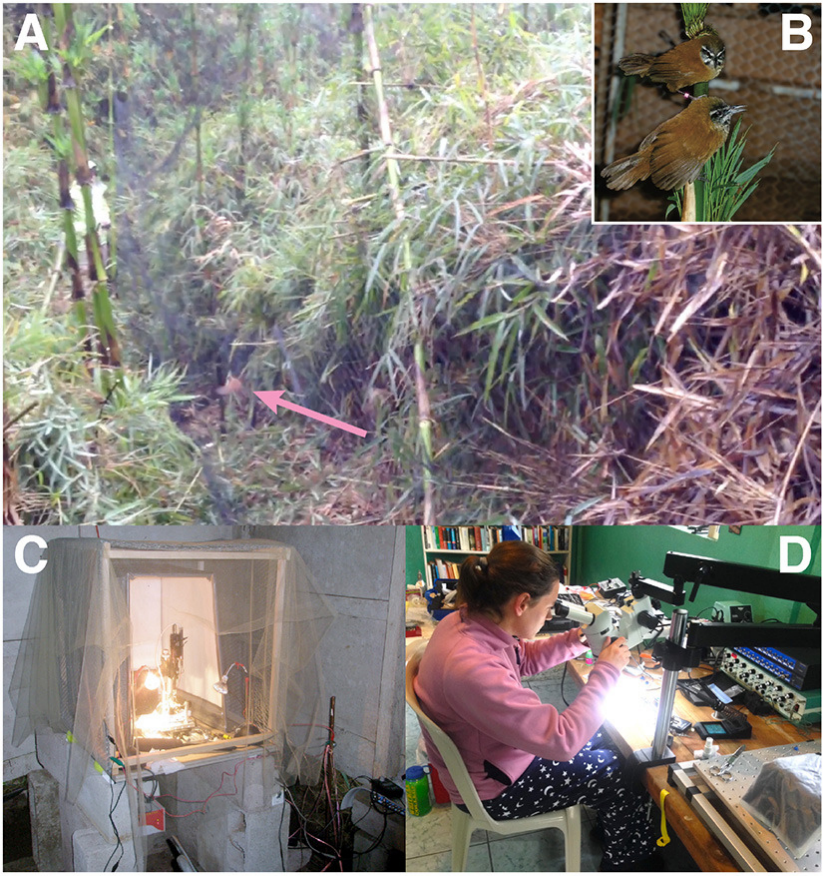
Capture and electrophysiological recording preparation. **(A)** A photograph of a plain-tailed wren (pink arrow) that was caught by a mist net. **(B)** A female (top) and male (bottom) plain-tailed wren in captivity. **(C)** The first electrophysiological rig that we used for recordings in urethane-anesthetized wrens. The vibration isolation table was a large tile plate that rested on tennis balls atop cinder blocks. A mesh was placed over the rig to prevent flying insects from interfering with the recordings. Note the copper rod used for grounding to the right of the rig. **(D)** Electrode arrays for chronic recordings were constructed at the field station in an improvised experimental rig–visible are the temperature controller (bottom right), amplifier and recording system (center right), and microscope mounted to a door used as the table. Both **(C,D)** are at the Yanayacu Biological Field Station and Center for Creative Studies.

We captured wrens and made electrophysiological recordings from HVC neurons in urethane-anesthetized animals (Figures 3A–C; Fortune et al., 2011). In these experiments, we presented short duration duet songs, songs that contained only the female or male syllables, and songs in which the inter-syllable intervals were digitally altered. Surprisingly, we found that neurons in both female and male HVC not only responded to their own syllables, as expected, but also to the other bird's syllables. Further, we found that neurons in HVC responded most strongly to the combined duet performance. That is, HVC neurons responded more to playback of intact duets than to the sum of the responses to the female and male components played separately (Fortune et al., 2011). This result suggested that, even though each bird only sings its part, the neural circuits controlling their singing integrate the combined output of the two birds. This was the first clue we had that the neural system for turn-taking spans both birds (see Figure 1B), as neurons responded best to the combined signal.

We also observed an interesting sex difference in HVC activity. HVC neurons in males responded more strongly to female syllables than to their own syllables (see Figure 3B in Fortune et al., 2011). In other words, HVC neurons in males responded more strongly to heterogenous cues than autogenous cues. In females, neurons responded more to autogenous cues (the female syllables) than heterogenous. In sum, neurons in both females and males responded more strongly to female syllables. This suggested that female syllables have more salience in the coordination of duet performances than male syllables. We interpreted this result that females provide the leading cues for coordinating duet performances. A similar conclusion was made in duetting bay wrens, based on behavioral results (Levin, 1996a,b).

What neural mechanisms give rise to the facilitated responses to duets in HVC? To understand the mechanisms by which HVC neurons integrate signals, we digitally manipulated either the female or male solo song by altering the gaps in between syllables (see Figure 4 in Fortune et al., 2011). For example, we modified or eliminated the gaps between syllables and discovered that stimuli with proper timing of syllables elicited significantly greater responses than stimuli that did not have proper timing. These data suggested the pattern generating circuit in each bird is tuned to the temporal structure of duet singing. Stimuli with normal gaps elicit stronger responses because the timing of sensory cues matches the temporal structure of the CPG. In contrast, stimuli with abnormal or missing gaps do not match the temporal structure of the CPG, resulting in weaker activation.

This finding may be a potential neural mechanism for a behavioral observation seen in duets recorded in the field. When females and males produce sequences of solo syllables, the temporal order of the syllables is similar to that when they sing duets, reflecting the role of a CPG in song production. Critically, when a bird (usually a male) drops a syllable, the partner often continues to sing its normal pattern of syllables. Interestingly, the inter-syllable interval is altered without the male, suggesting heterogenous input modulates the timing of the ongoing CPG (see Figure 1 in Fortune et al., 2011).

### Neurophysiological recordings from duetting wrens

Our initial experiments suggested that circuits in HVC integrate information from both birds (Fortune et al., 2011). However, these experiments were conducted in urethane-anesthetized birds, and it was unclear how auditory feedback from the partner influenced vocal production and turn-taking in awake, behaving birds. To understand the neural mechanisms of sensory-motor interactions underlying turn-taking we needed to record from birds when they were duetting. Knowing these experiments would be technically challenging in remote field sites, we decided to use multi-unit recordings (record from multiple neurons at one time). We also had to consider what type of equipment would be best to capture the neural activity in duetting birds. We decided to use a commercially available wireless recording system (Multi-Channel Systems MCS GmbH, Germany) for two reasons. First, wrens require bamboo in their cages which is incompatible with wired tethers. Second, wireless recording systems require less hardware that is also easier to implement at field sites.

We captured pairs of wrens on their territories (Figure 3A) at the Yanayacu Biological Field Station and maintained them for a short time (1–2 days) (Figure 3B) to allow them to adjust to captivity prior to surgery. Chronic electrodes were constructed in the field (Figure 3D) and were composed of four 50 micron wires with an additional reference and silver ground. The electrodes were implanted into either the left or right HVC. Once each bird recovered from surgery, we then captured the neural activity with a combined amplifier/digitizer that we attached to the electrode connector on the head of the bird. This amplifier/digitizer was powered by a battery that we placed on the back of a bird. One of the challenges we did not anticipate was designing a jacket for the battery. Our first attempts were using jackets created for zebra finches. However, we quickly realized these jackets did not work for the wrens who live in thick bamboo and were much more adept at using their legs and talons to remove the battery. We eventually designed a harness that secured the battery to the back of the wrens and did not impede their behavior.

**Figure 4:**
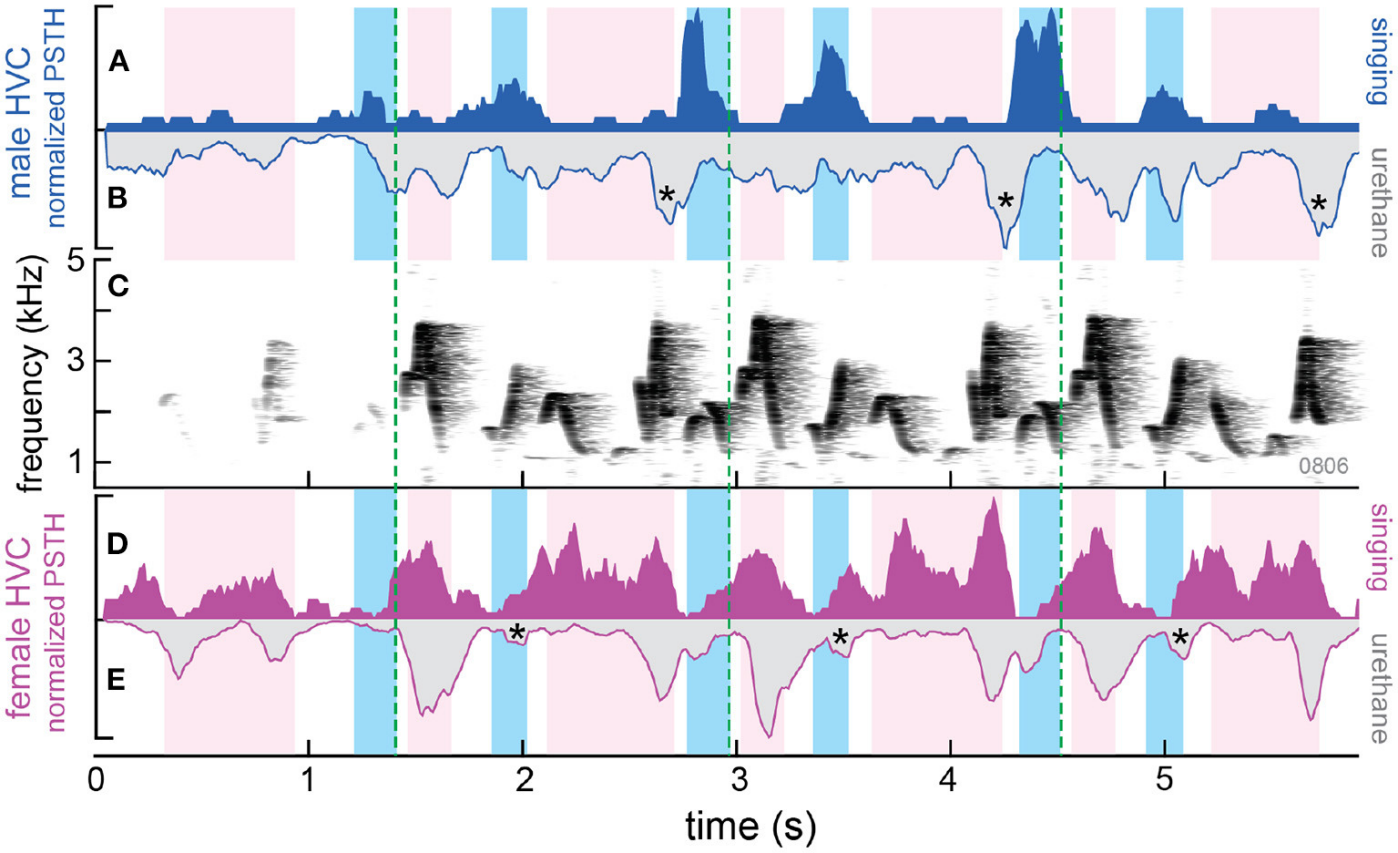
Differences in patterns of HVC activity in awake vs. anesthetized wrens. Background shading indicates the time each bird sang a syllable: darker blue for male and lighter magenta for the female. **(A)** Normalized PSTH shows HVC activity recorded from the male while duetting (singing; solid blue). **(B)** Inverted PSTH showing activity in response to playback (20 repetitions) of the same duet while the male was anesthetized with urethane. The histogram for activity during urethane anesthesia has been inverted to highlight the temporal relations in HVC activity when the bird is awake and singing and when the bird is anesthetized. Stars highlight increases in HVC activity near the end of partner (female) syllables. These increases in HVC activity may be inhibitory auditory responses in awake birds that are revealed by the action of urethane anesthesia. **(C)** Spectrogram of the duet produced by the pair of wrens. Dotted green lines highlight repetitions of duet motifs. **(D,E)** HVC neural activity in the female during singing and urethane as in **(A,B)**. Stars same as in **(B)**, but for recordings in the female.

Based on work in other songbirds and our previous finding in anesthetized wrens (Margoliash, 1986; Mooney, 2000; Mooney et al., 2001; Fortune et al., 2011), we expected an increase in HVC activity when each bird was singing (premotor activity) and an increase in HVC activity when each bird heard its partner (auditory-evoked activity). When we recorded from HVC of duetting wrens, we found that HVC activity in both the female and the male increased when they produced their own syllables, as expected (Figure 4, solid blue and magenta traces). However, we did not see an increase in HVC activity when either bird heard its partner's syllables. That is, there was an alternation of HVC activity that matched the alternation of syllable production in the pair (see also Figure 1A in Coleman et al., 2021).

Interestingly, this alternation of HVC activity was also found in duetting white–browed sparrow–weaver (Hoffmann et al., 2019). The alternation in HVC premotor activity does not explain how birds synchronize their vocalizations. How does heterogenous auditory feedback modulate the timing of vocalizations in partner birds? Surprisingly, a clue came from a comparison of results from experiments in awake and anesthetized wrens.

We used urethane as an anesthetic – urethane blocks GABAergic transmission (Accorsi-Mendonça et al., 2007). When the wrens were awake, HVC neurons were only active when the bird was singing its own syllables (premotor activity). However, under urethane anesthesia, HVC neurons, particularly in males, responded to playback of syllables from both birds (Figure 4, gray traces with blue and magenta outlines). This finding supported the idea that heterogenous activity in awake animals activates GABAergic circuits that inhibit premotor activity in HVC. Urethane blocks this inhibition so that when the birds are anesthetized, heterogenous auditory input is ‘unmasked' and now excites HVC neurons. Future experiments will more directly test the role of GABAergic inhibition in timing of turn-taking in wrens.

GABAergic inhibition has also been shown to coordinate another form of turn–taking in songbirds. In an elegant study, Benichov and Vallentin (2020) studied the neural basis for the coordination of calls between male zebra finches. Microinfusion of muscimol, a GABA agonist, resulted in a degradation of the timing of responses to calls. However, microinfusion of gabazine, a GABA antagonist, decreased the latency of responses to calls. This result contributes to the idea that turn–taking is an ancestral feature in songbirds and that inhibition may be a phylogenetically wide-spread neural mechanism for turn–taking. Odom et al. (2014) found that female singing is found in 71 percent of the species they surveyed across 32 families. A phylogenetic analysis of these data suggest that female song may be ancestral to oscine passeriform birds (Odom et al., 2014), a potential basis for turn-taking in these ancestral species.

The idea of inhibitory feedback between individuals is intriguing as it can help prevent overlapping vocalizations, a hallmark of turn-taking. Inhibitory feedback could also help explain the fast alternation of syllables. Once a neuron is inhibited, it can rebound from the inhibition (post-inhibitory rebound; PIR) and fire action potentials more quickly and at a higher firing rate. In addition, PIR may help explain the supralinear response to playback of duet song–HVC responded most strongly to the combined duet performance. Perhaps this supralinear response is due to post-inhibitory rebound. Inhibition also supports the model that the two individuals act as a single circuit (Figure 1B). In this model, each bird produces song through a pattern-generating circuit and the timing of the pattern is modulated by inhibitory feedback from the partner–much like a half-center oscillator (Marder and Calabrese, 1996; Calabrese, 1999).

Inhibition may be a neural mechanism used for the precise coordination of turn-taking in other species of duetting songbirds. For example, we hypothesize that duetting birds which have precise alternation of their vocalizations, like white–browed sparrow–weavers (Hoffmann et al., 2019) and canebrake wrens, (Rivera-Cáceres et al., 2016), may rely on inhibition. However, other duetting species whose duets are less tightly coordinated, like the black-bellied wren (Logue, 2007), may not use feedback inhibition and might rely on other neural mechanisms for coordination. This hypothesis, that inhibition emerges as a mechanism to improve the precision of timing of turn–taking, can be examined in additional comparative studies.

## Practical challenges to field work with plain-tailed wrens

Neuroethological studies conducted in an animal's native habitat allow the organisms to express a wider spectrum of its behavioral repertoire. The wider and more naturalistic expression of behavior allows scientists to address new questions concerning the control of behavior with greater confidence in the relevance of the findings. However, work at field sites introduce numerous experimental, practical, and scientific challenges that are reduced in laboratory settings. Lack of control, repeatability, implementation of technologies, access to technical support, and the enormous array of practical issues of working in remote uncontrolled habitats are serious barriers to scientific progress.

As expected, we faced biological and practical obstacles in establishing our research program using plain-tailed wrens. For example, a major hurdle in studying these birds is that they live in dense chusquea bamboo (Figure 2B). To capture the wrens, we discovered that we had to cut thin paths in the bamboo within their territories for our nets (Figure 3A). The nets themselves were constantly becoming tangled in the bamboo, and the birds, which navigate dense foliage every day, were well prepared to evade becoming tangled in the nets. The most successful approach for catching the wrens was to present a few playbacks of conspecific duets just after erecting the nets. Perhaps not surprisingly, the wrens were able to rapidly pin–point the locations of playback speakers, which we placed near the center of the net often about 0.5 m above the ground.

Another major hurdle is that our experiments rely on capturing both individuals of a female–male pair. The birds are monomorphic, making it difficult to distinguish during capture. We discovered that if the female was captured in the net first, the male would often simply depart. In contrast, if the male was captured first, the female would remain and was particularly aggressive in countersinging and exploring near the site of playback, facilitating her capture. Once in captivity, males tended to be more gregarious whereas the females tended to be more shy. For example, after capture, males readily accept worms and other food from experimenters hands, whereas females remained wary of humans.

Feeding the wrens was its own challenge. Wrens eat live insects, and we provided them with live crickets every hour. We were able to capture relatively small quantities of wild crickets by hand at our field site. Fortunately, there is a foundation near Quito, Wikiri, dedicated to frog conservation (www.wikiri.com.ec). Their efforts in frog conservation required that they farm crickets–which became the commercial source of the large numbers of crickets needed for our project.

Finally, wrens rely on nests in the bamboo to survive the cold nights (less than 12 degrees C) of their high–altitude habitats. To keep birds warm at our field sites that did not have heating, we placed our hot bead sterilizer (used for sterilizing surgical instruments) under a blanket that covered the cages at night.

As to more practical issues, electrophysiological studies require a truck-load of equipment that we had to transport to our field sites (Figures 3C,D). Of course, this equipment also requires electricity and, for our initial experiments, the electricity at our field station, the Yanayacu Biological Field Station and Center for Creative Studies, was generated from local hydro-power. The power depended on rain: the power would sometimes run out and so we had to implement a 12 V backup system using car batteries that were charged *via* solar panels.

Unexpectedly, the electrical grounding at our field sites was terrible to non-existent. For grounding, we purchased a 2 m copper rod (1.5 cm diameter) that we placed into the soil near our recording systems (see Figure 3C). Many of our initial experiments in anesthetized wrens were done in a shack that was open to the surrounding cloud forest. We had to protect the recording electrode from the many, many insects that were attracted to our lights, so we used a gauze-like material (chiffon) that we draped over the rig (Figure 3C).

Technical problems that are simple to solve in the laboratory are often more difficult to solve in the field. For example, to solve a saturation problem with our amplifier, the company advised us to send the amplifier back to them to change an internal setting. The solution we eventually used turned out to be simple. We had inadvertently created a small battery between our recording electrode and our reference electrode by using two different materials – our recording electrode was made from carbon and the reference electrode was silver. To solve this problem, we purchased a carbon art pencil, cut off the tip, and used Silverprint (GC Electronics, Rockford IL USA) to make it into a reference electrode. It worked like a charm.

Despite these challenges, we find field work personally and scientifically rewarding. Personally, solving problems in the field is enjoyable as it often requires a deeper understanding of the fundamentals of the tools and approaches that we use. For example, in the lab you might simply replace a part or send it for repair whereas in the field you often must devise alternative solutions. Scientifically, each species has evolved in its own environment and therefore has its own idiosyncrasies. These idiosyncrasies provide insights into, for example, how variability in nervous system control strategies is used to produce variability in behaviors. Further, biology requires a comparative approach to differentiate universal mechanisms from idiosyncratic features. Field studies are often the only avenue to study non-traditional species.

## Discussion

We believe the conceptualization of interacting animals acting as a unit is useful for the study of social behaviors in other species including duetting in Drosophila (LaRue et al., 2015; Coen and Murthy, 2016), singing mice (Okobi et al., 2019), antiphonal communcation in primates (Miller et al., 2009; Takahashi et al., 2013; Pika et al., 2018), and humans (Levinson, 2016). We found that the nervous system of plain-tailed wrens is ‘tuned' to the combined performance, therefore, thinking of cooperating individuals as a single unit may reveal neural mechanisms that are not obvious or present when thinking of animals as simple senders or receivers.

Future studies using plain-tailed wrens could focus on three major questions. First, How do males and females learn their respective parts? In other words, how do wrens of each sex learn the timing and identity of their own syllables? One possibility is that both female and male wrens learn both parts of the duet and then participate in duets as adults, in sex-specific manner. Alternatively, females only learn and sing female syllables. Gaining insights to song learning requires longitudinal recordings while young wrens hear adult models and then later develop their songs. One potential role of chorusing, in which several plain-tailed wrens simultaneously perform the same syllables (Mann et al., 2006), is in learning sex-appropriate roles in duet singing (Rivera-Cáceres et al., 2018).

Second, how do plain-tailed wrens learn to coordinate duet performances with their partners as adults? Anecdotally, when we caught female and male wrens from different territories, they sang together. Initially, the duet is not well-coordinated and very short (2–5 motifs), but after time the synchronization and the length of the song improves. One experiment to test this is to form new male/female pairings of wrens from distant territories or even from groups that sing different dialects. We could then quantify the changes in duet performances that emerge over time. Further, we predict an emergence of clear inhibitory responses in the brains of each wren as duet performances improves. Finally, what are the rules for duetting in plain-tailed wrens? Several studies in other species have described the behavioral rules and signals embodied in duet performances (Logue, 2006, 2007; Rivera-Cáceres et al., 2016; Rivera-Cáceres et al., 2018). Understanding these rules in plain-tailed wrens may facilitate future experiments that may reveal the roles of neural activity in HVC and other song nuclei in turn-taking.

## Author contributions

All authors listed have made a substantial, direct, and intellectual contribution to the work and approved it for publication.

## Funding

This study was supported by NSF IOS-1146792 (MC), NSF IOS-1146855 (EF).

## Conflict of interest

The authors declare that the research was conducted in the absence of any commercial or financial relationships that could be construed as a potential conflict of interest.

## Publisher's note

All claims expressed in this article are solely those of the authors and do not necessarily represent those of their affiliated organizations, or those of the publisher, the editors and the reviewers. Any product that may be evaluated in this article, or claim that may be made by its manufacturer, is not guaranteed or endorsed by the publisher.
