# Corrigendum: Neural mechanisms for turn-taking in duetting plain-tailed wrens

**DOI:** 10.3389/fncir.2022.1068385

**Published:** 2022-12-08

**Authors:** Melissa J. Coleman, Nancy F. Day, Eric S. Fortune

**Affiliations:** ^1^W.M. Keck Science Department, Claremont McKenna, Scripps and Pitzer Colleges, Claremont, CA, United States; ^2^Department of Psychology, Whitman College, Walla Walla, WA, United States; ^3^Department Biological Sciences, New Jersey Institute of Technology, Newark, NJ, United States

**Keywords:** antiphonal, birdsong, duets, central pattern generator, auditory feedback, neuroethology

In the published article, there was an error in affiliation 1. Instead of “W.M. Keck Science Department, Scripps and Pitzer Colleges, Claremont, CA, United States,” it should be “W.M. Keck Science Department, Claremont McKenna, Scripps and Pitzer Colleges, Claremont, CA, United States”.

The authors apologize for this error and state that this does not change the scientific conclusions of the article in any way. The original article has been updated.

